# Mycotoxins in milk for human nutrition: cow, sheep and human breast milk

**DOI:** 10.3205/id000021

**Published:** 2016-06-20

**Authors:** Herbert Hof

**Affiliations:** 1 MVZ Labor Limbach, Heidelberg, Germany

**Keywords:** mycotoxins, aflatoxin, ochratoxin, cow milk, human breast milk

## Abstract

Mycotoxins are produced pre harvest by some molds and secreted into various food items of plant origin, such cereals, vegetables, spices, coffee and nuts. If the food items are not stored under adequate conditions, a post harvest contamination may also occur. Animals and humans take them up by food items and some of them are stored and accumulated in different tissues and organs, so that food of animal origin may be contaminated, too. Especially aflatoxin and ochratoxin are secreted into milk by consumers of contaminated food. Since milk represents the major food source of newborns and infants, they are notably exposed to these mycotoxins. This health risk for these individuals may be of particular importance, because their ability to metabolize these fungal toxic agents is not yet fully developed at this stage.

## Introduction

In principle, fungi are able to challenge human health by various ways, namely by inducing infection, by triggering allergy or by intoxication (Table 1 (Tab. 1)).

The role of some pathogenic fungi as causative agents of infections is wellknown [1]. The allergenic potential of fungi is rather widely noted as well [2]. The pathogenic role of toxic effects of fungi [3], however, is highly underestimated.

There are at least 400 defined mycotoxins generated by certain toxigenic fungal species; but not every strain out of a toxigenic species will be able to produce toxins, which are secondary metabolites produced predominantly in the late-logarithmic phase of growth dependent on certain environmental conditions such as nutrient supply and temperature [3]. In addition, some volatile organic compounds from fungi may also be toxic for humans [4]. The effects of these biologically active substances may be quite different; some will be immunosuppressive [5] others carcinogenic, mutagenic, teratogenic as well as organ toxic for example for liver, kidney and nervous system; some mycotoxins exert rather hormone-like effects [3]. 

Only few of these fungal toxins are taken up by inhalation; the majority is ingested by various food items such as cereals, nuts, meat, spices, coffee, beer, wine as well as fresh fruit juices [6]. Acute signs of intoxication in humans are rare, because in general the amounts of toxins in the consumed food items are too low. But by accumulation the fungal agents may achieve gradually relevant concentrations in a human consumer, so that the detrimental effects will become manifest only after a certain lag-phase – sometimes after long periods. These chronic toxicities may be even accentuated by a cocktails of various mycotoxins present in food. Furthermore, the consequences may be aggravated by synergistic actions of other noxious agents such as chemicals (for example carcinogenic agents) or infectious organisms inducing chronic inflammatory reaction (for example Hepatitis B virus) [6].

To provide maximum security for public health the EU [7] has published regulations. Food items destinated for human use are in general controlled by governmental authorities; at least some aliquots are examined. Furthermore, there are regulations by EU authorities for certain mycotoxins in animal foodstuff [8]. Since the standards for animal nutrition are lower than for human use, animals ingest often feed with high levels of mycotoxins. Because the degradation of mycotoxins in the animals is limited, there is a carry over to humans by consumption of meat for example. Hence, the daily intake of various mycotoxins by such as deoxynivalenol, ochratoxin, zearalenone, fumonisin and patulin is considerable in an adult consumer. Especially in countries with low food hygiene the burden can be rather excessive [6], [9].

Some mycotoxins will be transferred into certain compartments of the consumer [10]. Of particular interest is the presence of mycotoxins in milk especially of aflatoxin and ochratoxin, so that already newborns may be exposed to these harmful agents [11]. 

The tricky problem is, that the consumer may not be able to recognize the risk, because mycotoxin producing fungi do not multiply in the milk itself and therefore the appearance is not different from innocuous samples; furthermore, the mycotoxins do not alter the taste of the food item. Hence, the content of mycotoxins in milk product should be assessed regularly in the laboratory or rules for the handling should be worked out. 

## The special role of aflatoxin

**Figure 1:**
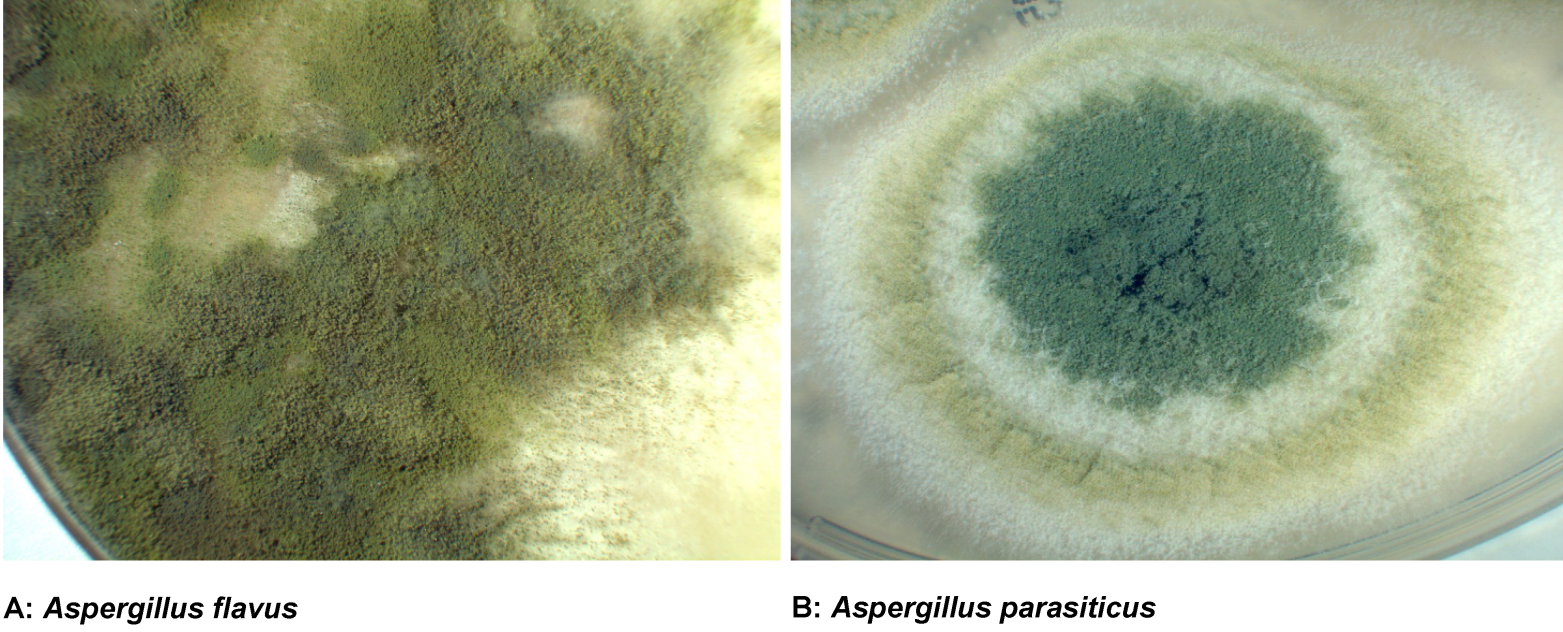
Cultures of *Aspergillus flavus* and *Aspergillus parasiticus*, respectively, both producers of aflatoxins, on Sabouraud Agar after incubation at 26°C for 7 days

*Aspergillus flavus* (Figure 1A (Fig. 1)) as well as other Aspergilli such as *Aspergillus parasiticus* (Figure 1B (Fig. 1)) are able to produce aflatoxin B_1_ as well as several other chemically related derivatives.

Aflatoxin B_1_ is generally found in various legumes and cereals, i.e. corn, wheat, rey, and barley. Especially under wet and warm environmental conditions the production increases; the contamination with mycotoxins may occur pre-harvest as well as post-harvest, when the storage conditions are favorable for fungal growth. In principle, the burden of aflatoxin is higher in tropical areas than in countries with moderate climate. Hence, the aflatoxin content in animal food grown in Europe is relatively low, i.e. under the limit of 4 µg/kg. Until recently, contamination was generally confined to imported foods. In 2013, however, because of the global warming, maize elevated in South-East Europe also contained considerable amounts [12]. Nuts, especially pistachios, almonds and hazelnuts, are often highly contaminated, so that the tolerable limit had to be raised to 10 µg/kg for ready to eat items [13]. In other food items, such as eggs and meat, the aflatoxin content is only low [10].

**Figure 2:**
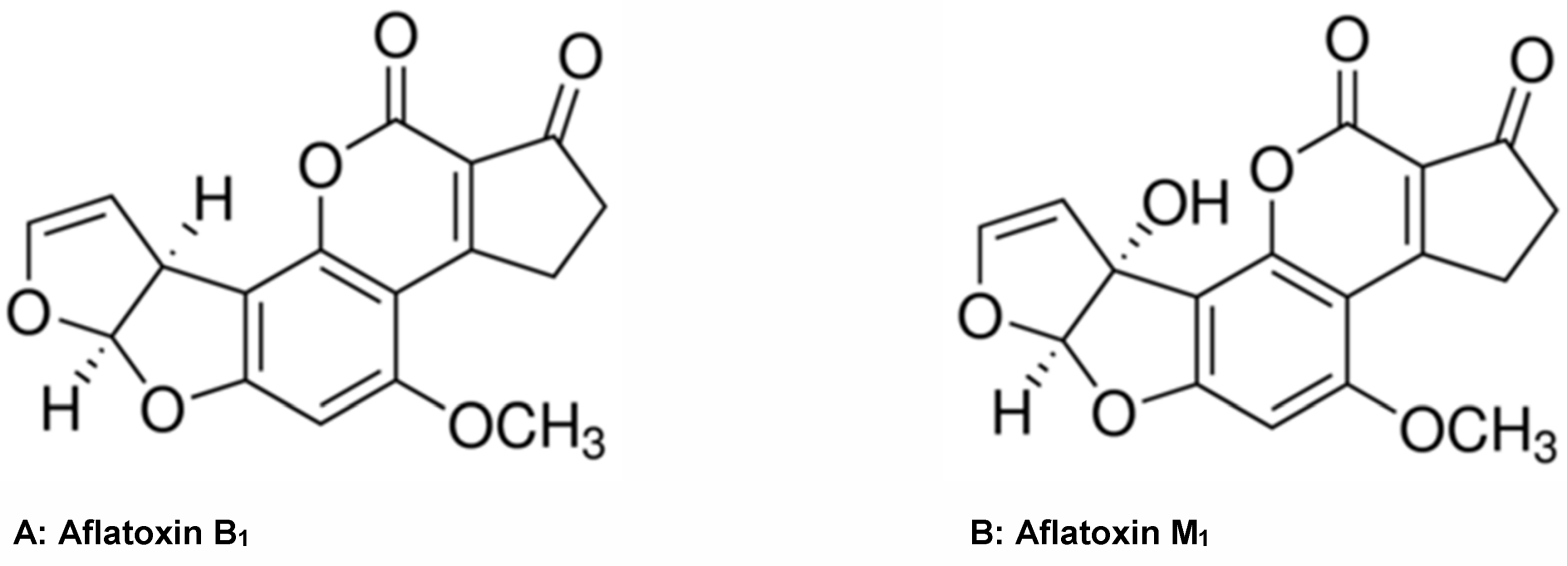
Conversion of aflatoxin B_1_ (found in food) to aflatoxin M_1_ in animals as well as humans (found in milk)

Aflatoxin B_1_ (Figure 2A (Fig. 2)) is highly carcinogenic and in addition immunosuppressive, mutagenic and teratogenic. In the liver it is converted through enzymatic hydroxylation by means of cytochrome P450-associated enzymes into aflatoxin M_1_ (Figure 2B (Fig. 2)), which about 10-fold less active than aflatoxin B_1_. It has been classified as a group1 human carcinogen [14]. This derivative is than excreted into the milk. The European Commission has determined the tolerable limit for aflatoxin M_1_ in raw milk, treated milk, and dairy products at 50 ng/kg. The concentrations in infant formulae, infant milk, and special food products should not exceed 25 ng/kg. Exposure of infants to aflatoxin M_1_ is worrisome, because their capacity for biotransformation of carcinogens is generally lower than that of adults and consequently they are more susceptible to adverse effects of mycotoxins [15].

## The special role of ochratoxin

*Aspergillus ochraceus* together with many other Aspergilli, and *Penicillium verrucosum* produce ochratoxins. Several derivatives have been described with ochratoxin A as the major agent. This fungal product is present in many food items of plant origin, i.e. cereals, legumes, nuts, spices, raisins, beer, wine and coffee. Since ochratoxin is not degraded in the intestine by a human consumer after ingestion, it can be found also in meat of pork and poultry. Consequently, this mycotoxin is generally present in many food items in large amounts. It is one of the the major mycotoxins in food. Furthermore, it has to be kept in mind that in most instances ochratoxin is detected together with other mycotoxins such as deoxynivalenol and zearalenone in the same food item [3].

Ochratoxin is accused to be carcinogenic, immunotoxic, neurotoxic and in particular nephrotoxic [6]. The metabolisation of this agent in humans is rather limited, so that it will be accumulated in the body. Its role in pregnant women has been assessed quite recently [16].

EFSA [17] fixed the “tolerable weekly intake” of ochratoxin A at 120 ng/kg equivalent to a tolerable daily intake of 14 ng/kg. (Other organizations have established even lower limits for intake of ochratoxin A). According to the consumption habits of a certain population this arbitrary limit can be often exceeded, for example by strong coffee drinkers [16]. For instance, in the so-called “mediterranean diet” one can find an 8-fold higher content of mycotoxins including ochratoxin [11].

## Mycotoxins in cow as well as ovine milk

At least some mycotoxins such as aflatoxin and ochratoxin are secreted into the milk, so that relatively high levels can be achieved. In contrast, other mycotoxins such as deoxynivalenol and zearalenone, are found in milk only in low concentrations [18]. This applies also to gliotoxin [19].

Dependent on the mycotoxin burden of animal food the concentrations of aflatoxin and ochratoxin found in cow milk may surpass in certain countries the tolerance limits [20], [21], [22], [23]. The maximum levels of aflatoxin exceeded the safety limits given by the EU, namely 0.05 µg/kg, in up to 75% [24]. Ochratoxin also may be present in concentrations much higher than the tolerable limits [25]. Since most of these fungal metabolites are highly temperature resistant [26], they may survive pasteurization and may be present in active form also in dairy products such as cheese and yogurt.

In ovine milk lower but not negligible amounts are found [27].

## Mycotoxins in human breast milk

In some regions of the world, the burden of food items with mycotoxins is substantial and therefore the oral intake of some mycotoxins by women notable. The content of mycotoxins in milk is related to the maternal dietary habits.

Especially in habitual consumers of bread, bakery products and cured pork meat ochratoxin is found often in breast milk even in considerable levels [25], [28], [29], [30], [31], [32], [33]. In Italy 74% of human breast milk samples yielded high amounts of ochratoxin; in certain samples very high concentrations of up to 405 ng/l have been detected [30] reaching almost the limits of 500 ng/kg proposed by the EU authorities [7].

The yield of aflatoxin M_1_ varies definitely. In tropical countries the burden seems to be very high in the majority of samples with levels far beyond the tolerated limits [34], [35], [36]. In some european and other countries aflatoxin M_1_ could not be detected or only in very few samples of human breast milk [15], [29], [37].

Furthermore, several other mycotoxins such as deoxynivalenol and zearalenone as well as some their metabolites could be found in human breast milk [38].

## Consequences

After birth, breast milk or infant formulas constitute an important or often sole food source for infants during their first months of life. It is evident that breast milk as well as infant formula diets represent a relevant source of mycotoxins for neonates and infants, since their presence in samples collected in several European countries has been documented. Therefore, the presence of mycotoxins in human milk is considered to cause certain risks for infant health [15]. In developing countries especially in countries with tropical climate, food for animals as well as humans is highly contaminated with mycotoxins; hence, the mycotoxin levels in breast milk as well as in infant formulas are in general definitely higher and hence proved to be a health risk for newborns and infants [9]. Because of the climate warming, it can be expected, that this risk may increase in the future in Europe, too. At least the exposition of animals to food contaminated with aflatoxin has become a relevant problem [12].

Form the various mycotoxins present in food aflatoxin and ochratoxin play the major role, because these fungal products are secreted into the milk of animals and women. Ochratoxin is the most abundant mycotoxin in milk. In European countries aflatoxin is still of minor importance. In general, aflatoxins included aflatoxin M_1_ [14] may exert adverse health effects for the exposed infant, namely growth retardation and jaundice [39].

Consequently, for the sake of protection of the consumers the risk assessment as well as the risk management of mycotoxins in milk products should be refined. The information about the various noxious agents and their respective health risks is the first step.

## Notes

### Competing interests

The authors declare that they have no competing interests.

## Figures and Tables

**Table 1:**
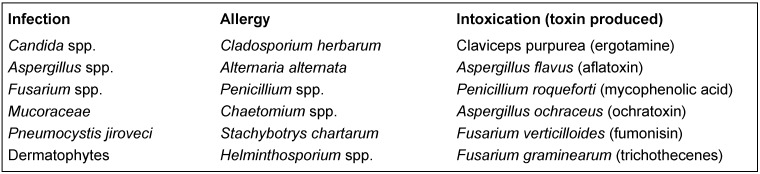
The role of some fungi as agents harmful for human health
